# Decision Making in Addictive Behaviors Based on Prospect Theory: A Systematic Review

**DOI:** 10.3390/healthcare10091659

**Published:** 2022-08-31

**Authors:** Javier Cabedo-Peris, Francisco González-Sala, César Merino-Soto, José Ángel Cahua Pablo, Filiberto Toledano-Toledano

**Affiliations:** 1 Departamento de Psicología Básica, Universitat de València, Avda. Blasco Ibáñez, 21, 46010 Valencia, Spain; 2 Departamento de Psicología Evolutiva y de la Educación, Universitat de València, Avda. Blasco Ibáñez, 21, 46010 Valencia, Spain; 3 Instituto de Investigación de Psicología, Universidad de San Martín de Porres, 15011 Lima, Peru; 4 Facultad de Ciencias Químico-Biológicas, Universidad Autónoma de Guerrero, Lázaro Cárdenas, El Centenario, Chilpancingo de los Bravo, Chilpancingo 39086, Mexico; 5 Unidad de Investigación en Medicina Basada en Evidencias, Hospital Infantil de México Federico Gómez National Institute of Health, Márquez 162, Doctores, Cuauhtémoc, Mexico City 06720, Mexico; 6 Unidad de Investigación Sociomédica, Instituto Nacional de Rehabilitación Luis Guillermo Ibarra Ibarra, Calzada México-Xochimilco 289, Arenal de Guadalupe, Tlalpan, Mexico City 14389, Mexico

**Keywords:** loss aversion, risk aversion, prospect theory, addictive behaviors, decision making

## Abstract

Traditionally, research on addictive behaviors has been based on the study of their risk factors, with impulsivity being the main risk factor. However, this study aims to approach this topic from the analysis of decision making. According to the prospect theory, low levels of loss and risk aversion will increase the probability of showing addictive behaviors. A systematic review of the possible relationships between these behaviors and prospect theory was carried out. To this end, the works that have studied loss and risk aversion in populations with addictive behaviors to date (N = 15) were compiled. Apart from other eligibility criteria, the selection process was only performed with studies that included the prospect theory or cumulative prospect theory, in English or Spanish, since 1979. WoS, Scopus, Dialnet and PsycInfo were the information sources selected. For this purpose, PRISMA guidelines have been followed. It was found that users of addictive substances show less loss aversion than nonusers. These results cannot be transferred to pathological gamblers. The significance of this work for future research and the implementation of prevention and intervention programs is highlighted. The results show an approach to addictions from a novel perspective.

## 1. Introduction

Addictive behaviors have been traditionally studied in relation to variable impulsivity. Coates et al. [1], Kale et al. [2] and Meikle et al. [3] are examples of this. Regarding addictions, having a high degree of impulsivity can be explained by two accounts that are not mutually exclusive, according to Verdejo-García et al. [4]. The first one states that these high levels of impulsivity are the product of the addiction itself, while the second one holds that impulsivity is a risk factor for becoming addicted. On the other hand, there are other more concise studies that point out that it is the automatic, although controllable, sensation of desire that triggers cravings when consuming certain substances, such as alcohol [5,6]. Impulsivity has also been correlated with cocaine consumption [7,8,9] in a greater manner than pathological gambling [10]. Furthermore, from a neuropsychological point of view [11,12], studies suggest that these differences in impulsivity and compulsivity are triggered by disruptions on the function of dopamine in the brain in the drug- and food-addicted population. Furthermore, impulsivity has been linked with alcohol use disorder patients who presented with high glutamate levels [13]. However, this brain disease model has faced critics due to its low practical benefits towards people who have exhibited addictive behaviors [14].

Moreover, these investigations leave aside the cognitive and rational aspects of behaviors. Other research has tried to approach these aspects. This is the case of Carmona-Perera et al. [15,16], who found utilitarian bias among alcohol- and polysubstance-dependent people while making decisions.

Kahneman and Tversky [17] coined the term prospect theory (PT), which states that each decision involving risk, regardless of how fast it is made or the perceived risk, is mediated by systems 1 and 2. System 1 responds to automatic cognitive processes, and system 2 responds to those that require concentration [18]. PT first appeared in 1979, although it was not until the work of Meyerowitz and Chaiken [19] that it was applied in the field of psychology per se. Addictive behaviors can be explained by this theory.

Human behavior usually has a rational basis, which some authors have tried to keep in mind when studying addictive processes [20,21]. This is the case with the work of Becker and Murphy [22], who explained that something becomes addictive when past consumption leads to future increased consumption, since utility is maximized. According to these results, a person who does not take the future into account will be more likely to engage in addictive behaviors. However, this theory does not consider the value that each individual gives to each behavior. In accordance with Tversky and Kahneman [23], people assign this value in terms of the perceived risk involved in the decisions they are about to make. Therefore, according to their approach, those who present greater loss aversion (LA) will be less likely to initiate behaviors that may generate negative consequences, as is the case for addictive behaviors. Tversky and Kahneman [23] define risk aversion (RA) as a bias, an error committed by system 1. This bias, when appearing before the onset of addictive behavior, becomes a protective factor, since the person will value possible health losses more negatively than possible gains.

Bickel and Marsch [24] conducted a review of studies of delay discounting and its relationship with impulsivity and loss of control in drug users. Nevertheless, the present paper aims to find the possible relationships of these behaviors with LA and RA, as was also proposed for future research in the abovementioned study. Some studies have explained the processes of addictive behaviors from the point of view of PT [25,26]. However, they did not consider the LA variable. In contrast, other works have studied this variable [27,28,29] in relation to addictive behaviors without taking PT into account. Some recent papers apparently confirm that addictive behaviors are related with LA, such as cigarette smoking and other substance use [30] and alcohol use disorder [31]. 

### The Current Study

The objective of the present article is to analyze the studies that have tried to measure the constructs of LA or RA according to PT among subjects engaged in addictive behaviors to date [17]. Our research question is whether those who engage in addictive behaviors show less aversion than those who do not. As a secondary objective, we seek to unify the results of this field to explore possible differences between drug addictions and behavioral addictions. Furthermore, the measurement tools that have been implemented to assess RA and LA will be further discussed.

## 2. Materials and Methods

A systematic review was carried out. The main topic of this review was the study of addictive behaviors and their relation to the cognitive processes defined by the PT of Kahneman and Tversky [17]. To do so, the preferred reported items for systematic reviews and meta-analyses (PRISMA) guidelines for systematic reviews were followed [32,33]. Prior to that process, a deep search of Cochrane was performed to ensure that no previous reviews had studied this topic. This systematic review was registered on the international prospective register of systematic reviews (PROSPERO) with the number CRD42022333455.

### 2.1. Search Strategy and Information Sources

Three search iterations of the databases and a manual search were carried out, due to the low number of documents that met the eligibility criteria. The first iteration included this combination of terms: (substance OR addic *) AND (“prospect theory” OR heuristic). During this first search, the term “substance” was found to be not very specific, since many documents related to the fields of mathematics, physics and engineering appeared. Thus, the term “addic *” was added to this formula to narrow the search for addictive behaviors. Accordingly, the inclusion of addictive behaviors not based on drug consumption was achieved. Gambling disorder is one example of this. The term “heuristic” has been used as a synonym of PT by some authors, so its inclusion in the search strategy was considered necessary.

The information sources used to perform this first iteration were the Web of Science (WoS), Scopus, Dialnet and PsycInfo. The results were refined to show all scientific articles and conference proceedings published between 1979 and 2020 (both included) written in English or Spanish. This procedure was repeated in all of the databases mentioned above, and 1979 was settled as the starting year, since it is the year in which PT was officially published for the first time [17]. Regarding PsycInfo, only human criteria were selected, as this is a function that this database provides.

Subsequently, a second iteration was carried out because of the perceived need to add a new term that appeared in different results obtained throughout the first iteration: “cumulative prospect theory” [34]. This theory is a derivation of PT that approaches the subject from the perspective of decision-making under risk and uncertainty. Therefore, it was interesting to add it to the study. The same databases as in the previous iteration were reviewed but using a new search strategy instead: (substance OR addic*) AND “cumulative prospect theory”. As was the case in the first iteration, only articles and conference proceedings written in English or Spanish between 1979 and 2020 (both included) were selected. Additionally, only human criteria were selected in PsycInfo. The inclusion of the new term “cumulative prospect theory” narrowed the results.

As was the case in the second iteration, a third iteration including the terms “loss aversion” and “risk aversion” was performed. The new search strategy was as follows: (substance OR addic *) AND (“loss aversion” OR “risk aversion”). The inclusion of the terms LA and RA came from the fact that they were both used indistinctly by the authors of the works found in the first two iterations. The results were broadened when using this new formula, and new studies appeared, even though duplicates were also returned.

Finally, a manual search of the references of the three previous iterations was carried out with the intention of identifying new eligible studies. For this purpose, the articles that were selected after conducting the screening of the three iterations were isolated, and their references were read to check their suitability for inclusion in the present study. The three iterations and the manual search were finished on 20 September 2021.

### 2.2. Eligibility Criteria and Selection Process

The screening process was performed according to these inclusion criteria: (a) studies of the relationship between PT and cumulative PT with addictive behaviors, (b) papers that included the terms LA or RA, (c) studies that measured LA or RA with assessment tools, (d) scientific articles and conference proceedings and (e) experimental studies using human samples. Exclusion criteria were (a) scientific articles that applied only a neurological test to measure the variables of LA or RA; (b) studies that included behaviors that, although considered addictive by their authors, ddido not involve drug consumption or pathological gambling (e.g., internet addiction, obesity, etc.); and (c) studies whose samples were not composed of high-level drug users or pathological gamblers after diagnosis or an assessment test.

This process was performed by two researchers independently and then combined until they arrived at a consensus. A third researcher supervised the results to confirm the quality of their task.

### 2.3. Ethical Considerations

This study is a part of the research project HIM/2015/017/SSA.1207 “Effects of mindfulness training on psychological distress and quality of life of the family caregiver,” which was approved on 16 December 2014, by the Research, Ethics, and Biosafety Commissions of the Hospital Infantil de México Federico Gómez, National Institute of Health, in Mexico City. While conducting this study, the ethical rules and considerations for research with humans currently enforced in Mexico [35] and those outlined by the American Psychological Association [36] were followed. All family caregivers were informed of the objectives and scope of the research and their rights according to the Declaration of Helsinki [37]. The caregivers who agreed to participate in the study signed an informed consent letter. Participation in this study was voluntary and did not involve payment.

### 2.4. Data Collection Process

To collect as much information as possible, the 15 articles were read in detail. Two researchers read and extracted the most-important data from each of the 15 articles to minimize possible bias. Then, they compared their answers until they arrived at a consensus. As was the case in the selection process, a third researcher revised their work to verify the quality.

These researchers extracted the data on publication year, authorship, study design, objective, sample, control group, methods, results and limitations. To be more precise, the objectives section explored the most-relevant objectives of each study in relation to the main topic of this systematic review. The sample category was filled in with characteristics related to the total number of subjects, age, gender, addictive behavior, and any other characteristic that could be interesting to consider. Regarding the control group, the same information as in the sample case was extracted. The methods section included the instruments or tests that were used in each single study. The results section included the most important results and conclusions related to the topic of this study. Finally, the limitations category was filled in with the most-highlighted limitations from each of the 15 studies.

## 3. Results

### 3.1. Study Selection and Study Characteristics

A total of 1191 published works were found in the first iteration: 678 on WoS, 311 on Scopus, 5 on Dialnet and 197 on PsycInfo. In the second iteration, 6 works were found: 2 on WoS, 1 on Scopus, 1 on Dialnet and 2 on PsycINFO. A total of 242 works appeared in the third iteration: 105 on WoS, 86 on Scopus, 1 on Dialnet and 50 on PsycINFO. In total, 1439 published works, both articles and conference proceedings, were found. Duplicates were screened with the tool RefWorks. A total of 1299 duplicates were identified and discarded. Therefore, 140 articles were sought for retrieval. Then, a manual search of those papers was performed, and 3 articles were considered appropriate for inclusion. These 3 articles were reviewed, and 1 of them was a duplicate, and the other 2 were included in the subsequent selection process. Thus, the final number of published works to be screened was 142.

**Figure 1:**
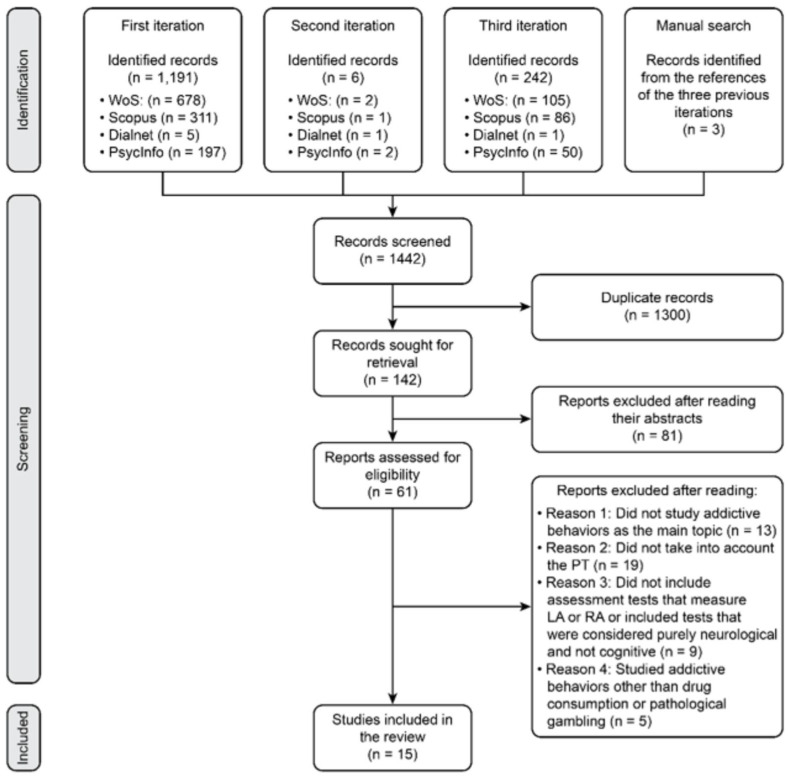
Flowchart of the study selection process according to PRISMA.

The eligibility of 61 papers was assessed after reading the abstracts of the 142 works that were previously selected. Some of the 61 articles were excluded after their body text was read in its entirety. The reasons for their rejection were not studying addictive behaviors as the main topic (n = 13), not take PT into account (n = 19), not including assessment tests that measure LA or RA, using tests that were considered purely neurological and not cognitive (n = 9) and studying addictive behaviors other than drug consumption or pathological gambling (n = 5). Finally, the review started with a total of 15 articles that fit the criteria (see Figure 1).

Information relative to the data collection results for each of the 15 articles included in this systematic review can be seen in Table 1. Of these 15 studies, 7 considered pathological gambling, 7 considered drug consumption and 1 considered both conditions, pathological gambling and alcohol consumption.

### 3.2. Results of Syntheses

According to the observed trend of the results extracted from this systematic review, there were no apparent differences between pathological gamblers and nongamblers when analyzing decision making. In only two papers [44,49] was a lower loss aversion observed in pathological gamblers. However, the work of Lorains et al. [44] found that this effect occurs only in pathological gamblers who are considered “nonstrategic” (e.g., gamblers of electronic gaming machines). Furthermore, in Genauck et al. [49], it is mentioned that this LA depends on the severity of gambling. In Ojala et al. [52], the results show that pathological gamblers tend to present a lower loss aversion than those in a control group when performing a loss task in a placebo situation. In contrast to these results, the study of Ligneul et al. [40] indicates that pathological gamblers exhibit greater LA than those in a control group. On the other hand, it is observed that the tendency for loss aversion may increase among pathological gamblers when they receive intervention treatment for more than 18 months [43].

When analyzing the articles containing samples of a drug-consuming population, differences were observed in LA between users and nonusers and LA was lower among users. This was maintained even after quitting [42] and in abstinent situations [48]. In the study of Meade et al. [50], the observed difference was even greater among those subjects who used cocaine and, in addition, presented an HIV diagnosis. Furthermore, those who were successful in quitting tobacco consumption exhibited greater loss aversion than those who were unsuccessful [39]. Regarding the study of Romanowich and Lamb [41], the cumulative PT was found to be a good explanation for the resumption of tobacco use, despite the contingency management that was used in the experiment.

The analysis of the instruments and tests that measure LA or RA in each study shows that none of them was used in more than one study, although they were quite similar. In terms of general methodology, no pattern distinguishing the studies focused on pathological gambling from those on drug consumption was found. It can be observed, however, that in most cases, the participants underwent tests prior to participating in the study to ensure that they met the diagnostic criteria of addictive behaviors. For example, 9 of the 15 studies used the DSM-IV [53] or the DSM-IV-TR [54]. According to a review proposed by Pellín et al. [55], the indiscriminate use of these manuals does not present notable differences that could influence the results. In most of these articles, they also used questionnaires or physiological tests to help identify such addictive behaviors or levels of consumption. The rest of the articles used tests to confirm the addictive behaviors of their subjects. Some of them used physiological tests, as is the case in the study of Romanowich and Lamb [41], which measured habitual tobacco use with the CO test (Vitalograph Inc., Lenexa, KS), and the study of Meade et al. [50], which used a urine test. The other four studies took their samples from addictive behavior treatment centers or associations, so it is assumed that the participants had been previously diagnosed. In the specific case of the study of Genauck et al. [49], alcohol consumers were diagnosed with the DSM-IV criteria [53], while a psychologist was trained to implement a screening instrument that helped to recognize pathological gamblers.

## 4. Discussion

Various studies have considered addictive behaviors from the standpoint of PT, which is considered a cognitive point of view. Thanks to this, considering new variables that could help understand the onset, maintenance and abandonment of an addiction has been possible. Most of these studies have been developed in the last decade, demonstrating a latent interest in this subject and a novelty of the field.

The results of this systematic review have been subdivided for two groups of addictive behaviors: pathological gambling (as a representation of behavioral addictions) and drug consumption. Pathological gambling can apparently be easily related to PT, likely because some specific actions that a person takes toward this practice may seem similar to daily decision making that contains some risk. In addition, the feeling of having power over the outcome to be obtained is intrinsic to pathological gambling, which is closely related to decision making in gain–loss situations. These are some possible reasons this behavioral addiction was studied in half of the articles that appear in this review.

Nevertheless, a strong relationship between pathological gamblers and LA was not observed in the overall results, possibly due to the lack of a distinction between “impulsivist gamblers” (low LA) and “emotionally vulnerable gamblers” (high LA), which was proposed by Takeuchi et al. [55]. They believe this distinction must be made, so they propose dividing the sample into these clusters to achieve a proper interpretation of the results. On the other hand, the work of Jantarakolica et al. [56] offers a different explanation for this lack of correlation. They considered a differentiation between loss and gain situations to be necessary, since gamblers modulate their LA based on the results obtained in a previous game.

Regarding drug consumption, high heterogeneity in terms of the analyzed substance was observed. This systematic review includes studies focused on heroin, tobacco, amphetamines, cocaine or alcohol users. Such heterogeneity hinders the generalization of the results. The overall results show that drug consumers are less RA than nonconsumers. These results seem to indicate that there could be an intrinsic factor that relates drug consumption to PT. Despite this, it is important to keep in mind that different drug addictions may not be compared with each other, although they have been taken together in this study. These results contrast with those of Breslin et al. [57], who openly stated that PT is not useful for explaining this phenomenon since alcohol does not influence decision making in gambling. However, the sample of this study was composed of social drinkers without a diagnosis of alcohol use disorder.

Two of the reviewed articles indicate that behavioral change can be learned better in loss situations [41,47]. This is an interesting idea to be applied in social interventions. On the other hand, this work is based on the premise that every drug consumer is aware of the risk that his or her consumption may entail prior to its implementation, so it can be related to their level of LA or RA. 

According to the European Monitoring Center for Drugs and Drug Addiction (EMCDDA) [58], the 29% of the European Union (EU) population aged 15–64 have consumed illegal drugs least once in their lives. This percentage is considerably different from one country to another. Sex differences were also shown, with men representing 60% of the total amount. Moreover, drugs have induced 16.7 deaths per million in the European Union from 2017 to 2020. This tendency has been increasing since 2012, exhibiting an increase of deaths from overdose of 82% of the population aged 50–65. Furthermore, 1.3 inhabitants per million have been diagnosed with HIV related to parental drug consumption. A tendency for the appearance of new psychoactive substances is easy to see. A total of 880 new substances have been reported since 2011, 52 of which appeared during 2021.

Regarding the latest Spanish Survey on Alcohol and Other Drugs in Spain (EDADES) [59], consuming cocaine or other illegal drugs once a month or less is the addictive behavior with the highest perceived risk. In the case of the Survey on Drug Use in Secondary Education in Spain (ESTUDES) [60], which targets only young people between 14 and 18 years of age, it is the habitual use of cocaine, heroin and ecstasy that shows the highest perceived risk. Both surveys agree that illegal substances are apparently the most dangerous. For that reason, one can consider illegal-drug consumers to present a lower RA in daily situations than the general population. The present results show an unequivocal trend of lower LA in illegal drug (heroin, amphetamines and cocaine) consumers. Nevertheless, it is not appropriate to compare these results with the results found among legal-drug (tobacco and alcohol) consumers due to the low number of articles found. Furthermore, the variable context must be considered, since the cultural social perception of each country and its legislation, as well as other aspects, will have a say on this.

Apart from that, EDADES [59] and ESTUDES [60] acknowledge that risk perception could act as a protective factor to avoid drug consumption. EDADES [59] shows that the slight decrease in tobacco consumption in recent years is inversely related to risk perceptions. A correlation between these variables cannot yet be assumed, and a deeper study must be performed. These two surveys asked their participants which method to mitigate drug consumption they thought to be more effective. Preventive actions such as school education, customs control and advertisement campaigns were three of the four most supported methods. Voluntary treatment for consumers was the other most highly valued method. In terms of preventive actions, information and an increase in risk perception were highlighted. This perception is difficult to operationalize due to its subjective nature, which can hinder intervention proposals [61]. However, according to the reviews performed by Erku et al. [62] and Goh et al. [63], health-risk messages aimed at smokers produced an intention for behavioral change among their samples. This suggests that further research on this topic is of great interest.

### 4.1. Future Research

Apparently, two different profiles of people with addictive behavior can be observed according to the LA variable: pathological gamblers and drug consumers. They are considered to be in the same field of “addictive behaviors”, but they present differences that could indicate a great distinction in their processes. Thus, different variables should be considered when studying them. In the same way, different treatment interventions should be proposed. Future research may be performed with the aim of enhancing the sample of this review to facilitate its generalization. On the other hand, determining the possible subgroups within the two proposed profiles is recommended. Possible examples of these subgroups can be constructed by dividing the sample by the substances they consume, the way they gamble and even the intensity at which they develop their addictive behavior.

### 4.2. Limitations

The main limitation of this review is the small number of studies found. Second, regarding the limitations of the articles themselves, there is a lack of consensus on the assessments and tests that were used. Third, it is difficult to determine the possible influence that LA has on addictive behaviors if their range is that wide. Fourth, screening based on the quality of the methodology of each study was not possible due to the small sample size. For example, articles were not refined in terms of measuring the onset of, increase in or abstinence from addictive behaviors. Furthermore, the sample includes studies that do not present a healthy control group or that present samples with more than one diagnosis (e.g., HIV). Apart from that, both the NIDA [64] and the EMCDDA [58] agree that addiction disorders show a high comorbidity with other mental illnesses such as depression and anxiety. Another limitation of this study is not taking it into account as a part of our eligibility criteria, as it can be responsible for affections in the decision-making process. 

### 4.3. Practical Implications

Attitudes, desires and intentions have a major impact on future behavior modification [65]. The present review may have some practical implications for intervening with people suffering from addictive behaviors. For example, it may help practitioners understand resistance and ambivalence in decision-making processes prior to change, as explained by the rationale for motivational interviewing proposed by Miller and Rollnick [66]. Narrowing the gap between health professionals and patients can help with the framing of decision making based on PT [23], which will, in turn, allow therapeutic-process usefulness to be assessed. This effect can improve treatment adherence [67].

Moreover, this research can help reduce the consequences associated with low RA, such as bad monetary management [68]. However, the scientific literature maintains the importance of taking as many variables as possible into account. For example, living through an economic crisis or boom has been shown to moderate economic decisions involving risk [69]. Therefore, the management of techniques that bring the subject as close as possible to a real situation to experience the negative consequences of addictive behaviors can be a good idea for intervention. Virtual reality, for instance, has great potential for drug-consumption treatment [70]. Guided visualization and covert sensitization are other examples of techniques approaching realism.

Regarding practical implications for researchers, there is a clear need for the use of a common instrument that measures LA or RA, in order to approach the easiest generalization of the results. For example, the recent appearance of the Loss Aversion Scale [71] can be considered a good candidate. However, it may need further adaptations and validation to other languages and samples. On the other hand, primary prevention on addictive behaviors directed to general population can implement PT on their messages, regarding LA or RA in the frame of gain versus loss situations. It has already been implemented on health messages [72] and smoking cessation [73].

## 5. Conclusions

This review is of great relevance for studying the relationship between addictive behaviors and PT due to its novelty. This is the first work that brings together the studies of this field that have been carried out to date. It is expected to have a great impact due to its practical implications. On the one hand, the inclusion of a rational decision-making paradigm, in contrast with the traditional current based on the study of impulsivity, may imply new possibilities and lines for prevention and intervention of addictive behaviors. On the other hand, there is a lack of consensus over the methodology that should be used to assess the constructs of LA or RA. It would be interesting to create a universal instrument that helps with this task.

## Figures and Tables

**Table 1:** Systematic information of the studies chosen for the review.

Authorship	Study Design	Objectives	Sample	Control Group	Methods	Results	Limitations
Blondel et al. (2007) [38]	Empirical research	To study decision-making under risk and with temporality in former heroin users.	N = 34. Age: $\bar{X}$ = 35; SD = 5.8 Former heroin users, under the effects of methadone. 62.86% male.	Yes. (N = 23 nonusers. Only men. Age: $\bar{X}$ = 37; SD = 10.4)	Blanqui game (7 items) and Stockholm game (14 items).	Consumers are less risk averse than nonconsumers. Both groups are similar in time.	The results cannot be generalized to other addictive behaviors since the study contemplates only one type (heroin use).
Ida et al. (2011) [39]	Empirical research	To analyze the relationship between risk aversion in a population trying to quit tobacco smoking.	N = 608. Successful smoking cessation: 321; 54,5% men; Age: $\bar{X}$ = 35. Failure to quit smoking: 287; 58.2% men; Age: $\bar{X}$ = 35.1.	No.	Associated analysis questionnaire, which offered alternatives to evaluate risk aversion, among other variables.	Successful quitters had lower loss aversion than unsuccessful quitters, both before and after 5 months of follow-up. There were no differences between the beginning and the end of the experiment within each group.	The cause of quitting smoking was not studied. The sample comprised people who managed to stop smoking for one week before joining the study.
Ligneul et al. (2013) [40]	Empirical research	To test the possible cognitive distortion of pathological gamblers in their perception of winning probabilities.	N = 18. Age: $\bar{X}$ = 33,2; SD = 11.5 Men, pathological gamblers.	Yes. (N = 20 men, nongamblers. Age: $\bar{X}$ = 31; SD = 7.3)	3 questionnaires: Barratt Impulsiveness Scale version 11, 40-item Sensation Seeking Scale and Gambling Attitudes and Beliefs Survey.	Greater loss aversion in pathological gamblers. Higher scores in attentional and motor impulsivity and in disinhibition and adventurousness.	Their exposure to pathological gambling behavior itself may affect their decision making. The choice of the sample is questionable. The experiment needs to be extended to other addictive behaviors.
Romanowich and Lamb (2013) [41]	Empirical research	To test whether the cumulative prospective theory is applicable to tobacco addicts. To test whether losses work better than gains for learning a behavior.	N = 25. Group of losses: 4% women. Age: $\bar{X}$ = 43; SD = 14. Group of gains: 5% women. Age: $\bar{X}$ = 39; SD = 13 Tobacco smokers, not intending to quit smoking.	No.	Smokers are divided into two groups: 1: they will lose $75 if they have smoked the day before a visit (they start with $375). 2: they will gain $75 if they have not smoked the day before a visit (they start with $0).	Consistent with cumulative prospective theory, the loss group quit smoking faster, but the gain group maintained it better over time.	The CO_2_ test is assumed to be reliable. There are differences between salary and the average number of cigarettes consumed per day in some participants.
Ahn et al. (2014) [42]	Empirical research	To test possible differences in the decision-making processes of consumers and ex-consumers.	n = 38 amphetamine addicts and detoxified (76.3% men. Age: $\bar{X}$ = 22.7; SD = 3.7). n= 43 “pure” heroin addicts and detoxified (81.4% male. Age $\bar{X}$ = 29.7; SD = 5). No gender distinction indicated.	Yes. (N = 48 nonconsumers. 79.2% men. Age: $\bar{X}$ = 24.7; SD = 4.9)	Iowa Gambling Task. Subsequently, their results were compared according to prospect valence learning with delta and decay learning rule and according to the value-plus-perseverance model.	Deficits in decision making were observed. Former heroin users had lower loss aversion and former amphetamine users had higher reward sensitivity than the control group.	The majority were male. Sociodemographic factors were not reported. Opioids and stimulants may be responsible for this decision making.
Giorgetta et al. (2014) [43]	Empirical research	To examine differences in the loss aversion of pathological gamblers and nongamblers, according to the stage of treatment of pathological gamblers.	N = 20 (17 were men). Age: $\bar{X}$ = 36.45; SD = 9.1. Pathological gamblers (n = 10 in treatment less than 6 months and n = 10 in treatment more than 18 months).	Yes. (N = 20 nonplayers; 17 are male. Age: $\bar{X}$ = 37.15; SD = 10.86)	A “decision under risk” task to assess loss aversion in gain and lose choices at 50% probability. In addition, the Baratt Impulsiveness Scale.	Pathological gamblers in treatment for more than 18 months presented greater loss aversion than the rest. There were no differences between the sample and the control group as a whole. Pathological gambling can be reduced with clinical treatment.	The sample was small. Pre and post measurements should be performed. Variables such as time discount or reward should be introduced.
Lorains et al. (2014) [44]	Empirical research	To observe differences in the decision making of the control group and the group of pathological players and between pathological players of strategic and nonstrategic games.	N = 39 (20 women). Age: $\bar{X}$ = 46.64; DT = 9.46. Pathological gamblers (n = 15 strategic gamblers; n = 24 nonstrategic gamblers).	Yes. (N = 41 nongamblers; 21 men. Age: $\bar{X}$ = 44.34; SD = 11.43)	Loss aversion task, which offered a 50% chance of winning or losing a variable amount of money. In addition, the Iowa Gambling Task.	Nonstrategic pathological players are less sensitive to losses. The nonstrategic group presented lower risk aversion than the control group and the group of strategic pathological gamblers.	The sample of pathological gamblers was seeking treatment. There was comorbidity with other mental disorders that may have affected their decision making but were not accounted for. The control group should present regular nonpathological gamblers.
Takeuchi et al. (2016) [45]	Empirical research	To test whether pathological gamblers differ from each other in terms of risk bias.	N = 31. Age: $\bar{X}$ = 33.4; SD = 7.5 Men, pathological gamblers, who have completed a cycle of 12-step therapy.	Yes. (N = 26 nongamblers. Age: $\bar{X}$ = 34.8; SD = 6.3)	Risky choice task.	Pathological gamblers should be divided into very aversive (emotionally vulnerable) or very aversive (impulsivist) groups for study, as there are significant differences between them.	Nonreal money was used. Some players were enrolled in an addiction treatment program.
Gelskov et al. (2016) [46]	Empirical research	To analyze the differences, at the cognitive and neuronal level between pathological gamblers and healthy subjects when making decisions.	N = 14. Age: $\bar{X}$ = 29.43; SD = 6.05. Men, pathological gamblers.	Yes. (N = 15 nongamblers, all of them were men. Age: $\bar{X}$ = 29.87; SD = 6.06)	A mixed play test that depended on whether the gain or loss situation came before or after, while performing an fMRI.	There were no significant differences in loss aversion between groups, but there was a tendency for pathological gamblers to have lower values.	Small sample. Pathological gamblers came from a treatment center. Events were presented quickly, which avoided delays between choices.
Strickland et al. (2017) [47]	Empirical research	To assess loss aversion in active cocaine users.	N = 38. Age: $\bar{X}$ = 45.7; SD = 5.8 All of them were cocaine users. 42% women. At the time of the experiment, they were not under the effects of this drug.	No.	3 questionnaires: valuation task, mixed gambles task and risk aversion task. They were given 30 dollars to carry out the experiment. At the end of the experiment, they received 10 dollars.	Less loss aversion among cocaine users. A rigid aversion to loss can generate a poor choice regarding cocaine consumption. Hence, being sensitive to this during treatment can help reduce drug consumption.	There was no control group. The possible influence of sociodemographic factors was not tested.
Bernhardt et al. (2017) [48]	Empirical research	To study probability discounting and loss aversion in alcohol consumers.	Study 1: N = 198. Age: $\bar{X}$ = 18.38; SD = 0.2 Men. Social alcohol users. Study 2: N = 114. Age: $\bar{X}$ = 44.77; SD = 10.56 18 women and 96 men, diagnosed with alcohol use disorder.	Study 1: No. Study 2: 17 women and 81 men. Age: $\bar{X}$ = 43.75; SD = 10.86	Two studies: Study 1: Value-based decision making for the study 1 sample. Study 2: Value-based decision making for the study 2 sample and the control group.	Study 1: Loss aversion does not predict changes in alcohol consumption. Study 2: Lower loss aversion in abstinent alcohol users. Subjects’ attitudes toward risk and loss make them more likely to relapse into binge drinking.	The used method needs to be verified. The rewards were of little value. Consumers were seeking treatment. Little female representation.
Genauk et al. (2017) [49]	Empirical research	To test, at a neuronal and cognitive level, whether people with alcohol use disorder and pathological gamblers show less loss aversion than a healthy population.	N = 34. n = 19 men, pathological gamblers. Age: $\bar{X}$ = 32.9; SD = 10. n = 15 men, diagnosed of alcohol use disorder. Age: $\bar{X}$ = 45.4; SD = 10.2.	Yes. (N = 17, nongamblers, neither alcohol users, all of them were men. Age: $\bar{X}$ = 38.8; SD = 11.5)	Both the loss aversion task, which asks about desire to gamble, and a fMRI.	The subjects showed a lower aversion to loss than the healthy control group, both cognitively and neuronally. Pathological gamblers showed differences in severity, with more severity resulting in less aversion.	The sample was small and only included men. People with comorbidities were not included in the sample.
Meade et al. (2018) [50]	Empirical research	To analyze decision making in cocaine users and HIV-diagnosed individuals independently and in combination at the cognitive and neural levels.	N = 69 (47 men). Age: $\bar{X}$ = 44.13; SD = 8.08. 16 non-HIV-positive cocaine users and 15 HIV-positive cocaine users. 21 non-HIV-positive cocaine users and 17 HIV-positive cocaine users.	No.	A loss aversion task and an fMRI administered at the same time.	Cocaine users have lower loss aversion, which is even lower among those with HIV. Neurologically, it seems that the pathways responsible for such aversion differ between cocaine users and people with HIV.	Subtle differences between groups could not be measured. Loss and gain situations were studied separately and connections between them were not analyzed. Cocaine users may have used other “soft” drugs.
Ring et al. (2018) [51]	Empirical research	To study risk perceptions in gain and loss situations, probability weighting and the level of loss aversion of pathological gamblers.	N = 48. Total mean age (sample and control group) is 38.9; SD = 14.7. Pathological gamblers group (n = 25; 3 are women). Habitual gamblers group (n = 23; 4 are women).	Yes. (N = 26; 6 are female. Played at most less than once a month).	4 tests: Risk elicitation task, time preference elicitation task, threat-of-shock task and fMRI.	No significant differences were found in loss aversion. Pathological gamblers took greater risks and were less sensitive to changes than those in the control group. No significant differences were found in the shock task.	Higher stakes were not measured. Only two observations were used to assess loss aversion. The money was not real. No sociodemographic differences were studied. The sample was very small. The isolation effect cannot be assumed to be completely true.
Ojala et al. (2018) [52]	Empirical research	To observe whether blocking dopamine D_2_/D_3_ receptors decreased probability-weighting biases in gain and loss situations in a sample of pathological gamblers and nongamblers.	N = 16. Men, pathological gamblers (age: $\bar{X}$ = 35.8; SD = 8.8).	Yes. (N = 21, men, nongamblers. Age: $\bar{X}$ = 32.1; SD = 11.4).	An equivalent certainty task in decision making. The entire group performed the task 2 times, at least 1 week apart, once after taking the placebo and once after taking a dose of dogmatil 400 mg (which acts as a dopamine D_2_/D_3_ receptor antagonist).	No differences between groups were found, except in the loss situation, wherein the pathological gambler group showed devalued loss probabilities compared to the nongambler group in the placebo situation.	The sample was small and composed exclusively of men. Moderate test–retest reliability in decision making in addictive behaviors. Individual risk perceptions varied substantially between tests.

## Data Availability

The raw data supporting the conclusions of this article will be made available by the authors, without undue reservation.

## Abbreviations

PT	Prospect theory
LA	Loss aversion
RA	Risk aversion
PRISMA	Preferred reported items for systematic reviews and meta-analyses
PROSPERO	International prospective register of systematic reviews
WoS	Web of science
EMCDDA	European monitoring center for drugs and drug addiction
EDADES	Encuesta sobre alcohol y otras drogas en España
ESTUDES	Encuesta sobre uso de drogas en enseñanzas secundarias en España
